# Functions of the Pancreas

**Published:** 1868-12

**Authors:** 


### ARTICLE VI.

### *Functions of the Pancreas.*

Notwithstanding the numerous and careful investigations that have of late years been directed to the elucidation of the functions of the pancreas, the recent researches of Kuhne, Fudakowski, and of our own countryman Dr. M. Foster, show that much still remains to be learned. It is now very generally admitted that the pancreatic juice acts upon the three important alimentary groups included under the heads of the Albuminous, the Oleaginous, and the Farinaceous. It converts all the albuminous or proteic compounds into a substance which is not coagulated at the boiling temperature, and possesses a high degree of diffusibility, termed peptone; it emulsifies fat more perfectly than any other secretion in the body; and, lastly, it exerts an amylolytic power, as Dr. Foster terms it, over starch, rapidly converting it into grape sugar or glucose. What is it that enables this clear, viscid, transparent fluid to effect such wonderful changes? Is the active agent single, or is it multiple? And what is the nature of the action in each instance? Is it analogous to the agency of a ferment—catalytic action—so that a minute portion of the juice will alter an indefinite quantity of the substance acted on; or is it a simple case of chemical combination?

Some progress has been made in replying to these questions. In the first place, we may refer to Kuhne's observations. It is generally stated in the text-books that lucin and tyrosin, substances that are well known to result from decomposition of albuminous compounds, are constituents of the pancreatic tissue, and are eliminated in the secretion. Kuhne shows that they probably result from the action of the juice on the peptone which has already been formed from albumen or other proteic compound. This is a matter of considerable interest; for it shows that already, while the food is still in the intestinal canal, a portion of it undergoes decomposition—a true luxusconsumption—into substances that are ordinarily regarded as belonging to the products of regressive metamorphosis. And it is also remarkable that the active agent of the pancreatic juice can effect, at a temperature of 100 F., the same changes in albumen as may be accomplished without the body by the addition of an acid and the application of a boiling temperature. It is probable that the substance termed Pancreatine is that to which this power is to be attributed. We may just add the result of one experiment: 100 parts of dry fibrin yielded 61 parts of peptone, 3.86 of tryosin, 9.1 of leucin and 26 parts of unknown substances, amongst which were anilin-like bodies, giving a red or violet color with chlorine water, or chloride of calcium.

Dr. Foster,s researches have been chiefly directed to the determination of the nature of the substance by which starch is converted into sugar. This amylolytic power, though commonly stated to be possessed by all albuminoid compounds at a certain stage of decomposition, is in reality limited to certain fluids; and these manifest it as well when fresh as during any subsequent stage of decomposition. Thus human blister fluid, the vitellin of the fowl's egg, and the serum of sheep's blood, are powerless upon starch when fresh, and equally powerless in all stages of decomposition. But, from Dr. Foster's investigations, it seems doubtful whether this remarkable power is really associated with any of the ordinary forms of albumen; it is certainly not with pancreatine, since, as he has shown, if pancreatic juice or infusion of pancreas be saturated with sulphate of magnesia and filtered, the whole of the so-called pancreatine is retained on the filter, and yet the fluid which has passed through the filter will be found to have lost a fraction only of its amylolytic power. In the same manner it may be shown, in the case of saliva, that the converting power depends neither on the mucus nor on the globulin, since it retains its energy when freed from both of these substances. The ferment, therefore, seems to be something quite separate and distinct from albuminous compounds. And this view is materially corroborated by the facts that the amylolytic power is by no means commensurate with the quantity of proteids present, and that the presence of neutral salts, even to saturation of the solution, has no appreciable effect. It is remarkable, however, that it should always occurs in fluids containing proteic or albuminous compounds, and that it should lose its powers at about the same temperature as effects the coagulation of the various albuminous compounds. It has been proved by Dr. Foster that in the case of saliva, and therefore we may with all probability assume in the case of pancreatic juice, the ferment is *not* consumed during its action on starch; which is fully borne out by the well-known fact that a definite quantity of amylolytic will sooner or later convert any quantity of starch into sugar. Dr. Foster has made a few additional investigations on the distribution of the amylolytic ferment, and has found it to exist in the tissue of the liver, in the pleural and peritoneal fluids, and in the blood and urine. He conceives that the disease diabetes is due, not to any excess, but rather to some modified action of ferment; since the amount of ferment passed in the urine per diem in six cases in which he examined it, in no way exceeded that passed by persons in health, and further, the blood was not found to be more amylolytic than in health. These experiments we hold to be in the right direction for affording an explanation of this singular disease, and we trust Dr. Foster will be induced to continue them.—*Lancet.*

